# Vascular Birthmarks as a Clue for Complex and Syndromic Vascular Anomalies

**DOI:** 10.3389/fped.2021.730393

**Published:** 2021-10-07

**Authors:** Andrea Diociaiuti, Guglielmo Paolantonio, Mario Zama, Rita Alaggio, Claudia Carnevale, Andrea Conforti, Claudia Cesario, Maria Lisa Dentici, Paola Sabrina Buonuomo, Massimo Rollo, May El Hachem

**Affiliations:** ^1^Dermatology Unit and Genodermatosis Unit, Genetics and Rare Diseases Research Division, Bambino Gesù Children's Hospital, IRCCS, Rome, Italy; ^2^Interventional Radiology Unit, Department of Imaging, Bambino Gesù Children's Hospital, IRCCS, Rome, Italy; ^3^Craniofacial Centre-Plastic and Maxillofacial Surgery Unit, Bambino Gesù Children's Hospital, IRCCS, Rome, Italy; ^4^Department of Pathology, Bambino Gesù Children's Hospital, IRCCS, Rome, Italy; ^5^Department of Neonatal Medicine and Surgery, Bambino Gesù Children's Hospital, IRCCS, Rome, Italy; ^6^Laboratory of Medical Genetics, Department of Laboratories, Bambino Gesù Children's Hospital, IRCCS, Rome, Italy; ^7^Medical Genetics Unit, Bambino Gesù Children's Hospital, IRCCS, Rome, Italy; ^8^Rare Diseases and Medical Genetics Unit, Bambino Gesù Children's Hospital, IRCCS, Rome, Italy

**Keywords:** vascular birthmarks, vascular anomalies, vascular tumors, vascular malformations, complex vascular malformations

## Abstract

Vascular birthmarks are common in neonates (prevalence: 20–30%) and mostly incidental findings sometimes with spontaneous regression (salmon patch and nevus simplex). Capillary malformations are found in about 1% and infantile hemangiomas are found in 4% of mature newborns. Vascular malformations are classified according to their most prominent vessel type. The term “capillary malformation” (port wine stain) includes a wide range of vascular lesions with different characteristics; they may be isolated or part of specific syndromic conditions. Part of the infantile hemangiomas and of the vascular malformations may require treatment for functional or cosmetic reasons, and in rare cases, investigations are also necessary as they represent a clue for the diagnosis of complex vascular malformation or tumors associated with extracutaneous abnormalities. Complex vascular malformations are mostly mosaicism due to early somatic mutations. Genetic advances have led to identify the main pathogenic pathways involved in this disease group. Diffuse capillary malformation with overgrowth, Klippel–Trenaunay syndrome, CLAPO syndrome, CLOVES syndrome, and megalencephaly-capillary malformation belong to the PIK3CA-related overgrowth. Capillary malformation–arteriovenous malformation underlies a fast-flow vascular malformation, sometimes manifesting as Parkes–Weber syndrome. Recognition of these different types of capillary vascular stains is sometimes difficult; however, associated findings may orient the clinicians while genetic testing may confirm the diagnosis. Lymphatic malformation frequently manifests as large masses that compress and/or infiltrate the surrounding tissues, representing a neonatal emergency when airways are involved. Infantile hemangiomas may cause functional and/or permanent esthetical damage, depending on their localization (such as periorbital area, lip, nose); large (more than 5 cm) infantile hemangiomas with a segmental distribution can be associated with obstruction or malformations of the underneath organs with complications: PHACE syndrome, LUMBAR/SACRAL syndrome, and beard infantile hemangioma. In our review, we discuss controversies regarding the international classification and emerging concepts in the field of vascular anomalies. Finally, we discuss potential developments of new, non-invasive diagnostic techniques and repurposing of target therapies from oncology. Complex and/or life-threatening vascular tumors and malformations are extremely rare events and they represent a considerable therapeutic challenge. Early recognition of clinical signs suggestive for a specific disease may improve therapeutic outcomes and avoid severe complications.

## Introduction

Vascular anomalies comprise a wide spectrum of disorders ranging from benign transient manifestations to complex and life-threatening diseases. The classification by the International Society for the Study of Vascular Anomalies (ISSVA) distinguishes vascular tumors from malformations (1). Tumors are subdivided into benign, locally aggressive, and malignant. Vascular malformations are defects in vasculogenesis, while vascular malformations are classified based on the basis of the most prominent vessel type (capillary, lymphatic, and venous). They also may be combined if composed by multiple vessel subtypes and may be associated with others anomalies.

ISSVA classification integrating genetic recent advances together with clinical phenotypes led to correctly identify clinical entities and avoid use of improper terms and consequent inadequate management. However, emerging diseases are not yet included in the current classification and there are some criticism about the use of some terms, such as “capillary malformation.” In addition, this classification is not yet adopted for liver and cerebral vascular anomalies.

Vascular birthmarks are common in neonates with a prevalence >30% (2) and may be undervalued by physicians. Indeed, even if the vast majority are transient or benign cutaneous manifestations (salmon patch, nevus simplex, and stork bite), in some cases, they may represent an important clue for the diagnosis of complex and syndromic vascular anomalies: vascular malformations and vascular tumors associated with extracutaneous anomalies.

Capillary malformations (CMs) are simple slow-flow vascular malformation. However, when localized on the forehead, they may be part of the Sturge–Weber syndrome (SWS). The association of overgrowth with CM belongs to the PIK3CA-related overgrowth syndromes (PROS). In addition, CMs have been described in capillary malformation–arteriovenous malformation (CM-AVM), although in this case, they are not slow-flow vascular malformations.

Cutaneous venous malformations (VMs) are slow-flow VMs. They are frequently single lesions but may be multifocal or associated with extracutaneous localizations, most frequently on the gastrointestinal tract.

Lymphatic malformations are slow-flow malformations of lymphatic vessels. They may be present at birth as voluminous masses in particular located on the head and neck, or manifest later.

Infantile hemangiomas (IHs) are the most common benign tumors in infancy with a prevalence of 4–5% (3). They usually present within the first weeks and are characterized by rapid growth in the first 4–9 months generally followed by spontaneous involution within 4–7 years, with or without scars. However, investigations and early treatment are needed in 12% of cases due to functional or permanent esthetical damage. In addition, large (more than 5 cm) IH may be associated with extracutaneous malformations. This is the case of PHACE and PELVIS/LUMBAR/SACRAL syndrome.

Although complex and/or life-threatening vascular tumors and malformations are rare diseases, they usually present at birth or in the first weeks of life. Therefore, even minimal skin signs suggestive for these conditions should be taken into account in order to achieve the correct diagnosis, to prevent future complications, and to improve the quality of life of the patients and their families.

## Materials and Methods

Review of the literature of the last 15 years has been made on PubMed using the following terms: “capillary malformation,” “PROS,” “Sturge Weber,” “Klippel-Trenaunay,” “CLAPO,” “CLOVES,” “megalencephaly-capillary malformation,” “lymphatic malformations,” “infantile hemangioma,” “PHACE,” “PELVIS,” “SACRAL,” “LUMBAR,” “venous malformations,” “blue rubber bleb nevus syndrome,” and “Kasabach–Merritt phenomenon.” Results have been checked by two independent authors in order to exclude duplicate papers and to select the ones to read *in extenso*. Every author contributed to write the parts of the manuscript related to her/his specialty.

## Results

Since vascular birthmarks are very common and mostly benign, they may be overlooked by the neonatologist. In rare cases, these lesions are a sign of complex vascular disorders. Moreover, vascular lesions are not always present or fully developed at birth and they may manifest during the first years of life. Thus, follow-up is essential to guarantee the correct diagnosis and to avoid misdiagnosis of complex diseases.

### Capillary Malformations

CMs, the most frequent vascular malformation, are slow-flow vascular lesions constituted by dilated capillaries, arterioles, and post-capillary venules. They are frequently simple and isolated. However, CMs may be part of syndromic disorders.

**Figure 1:**
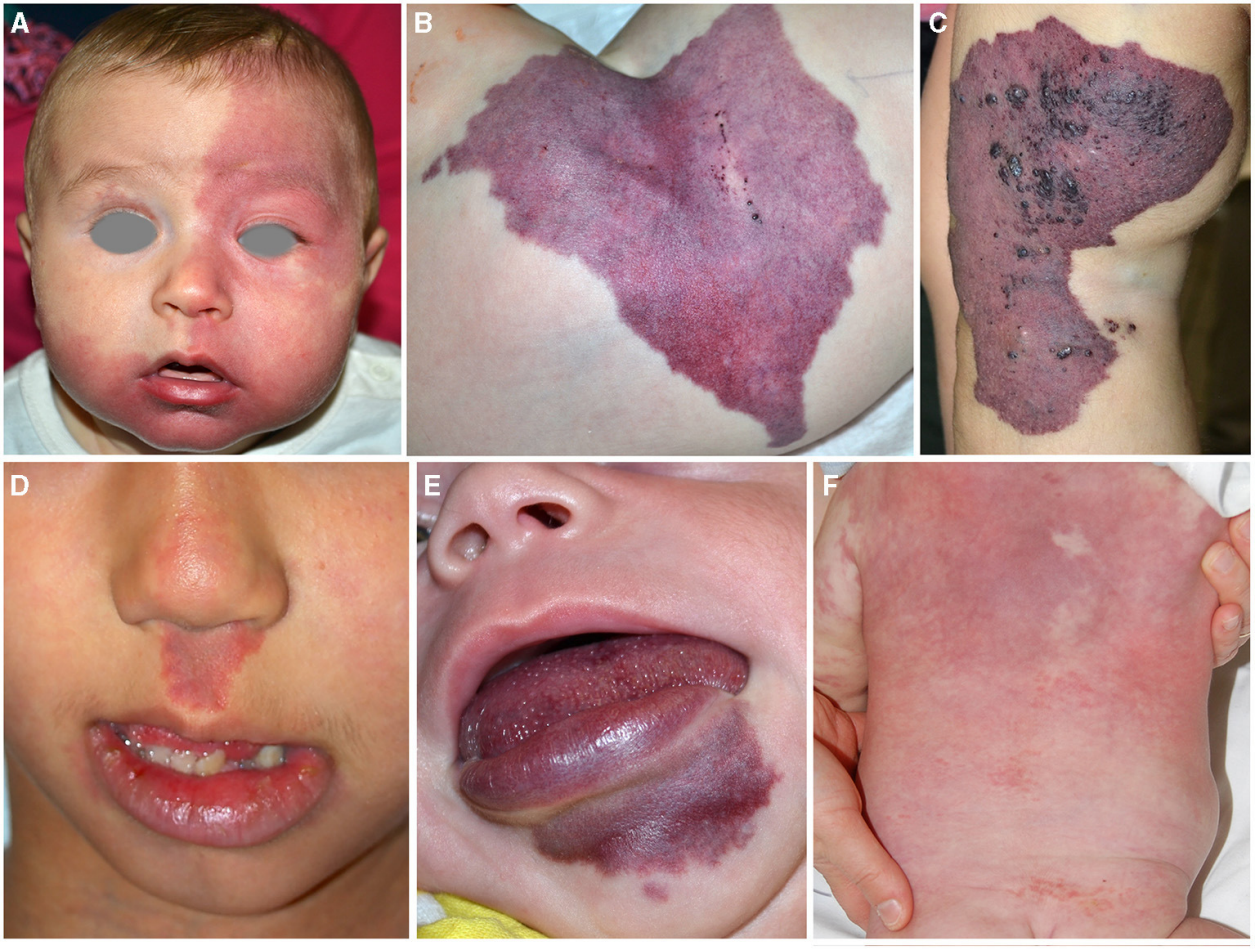
Capillary malformation involving the frontal area in an infant affected with Sturge Weber syndrome **(A)**. Violaceous patch of the hip in an infant affected with CLOVES syndrome. Crusted vesicles are present on the surface of the stain indicating the presence of a lymphatic component **(B)**. Capillary–venous–lymphatic malformation of the thigh in a child affected with Klippel Trenaunay syndrome **(C)**. Capillary malformation of the philtrum in a child affected with megalencephaly-capillary malformation **(D)**. Capillary malformation of the lower lip in an infant affected with CLAPO syndrome **(E)**. Reticulated and diffuse capillary malformation with blurred margins on the trunk in a 3-month-old infant affected with diffuse capillary malformation with overgrowth **(F)**.

*Sturge–Weber syndrome (SWS)*. Cutaneous CMs of the face associated with leptomeningeal and/or choroidal involvement define SWS. In particular, CM localized in the forehead, delineated at its inferior border by a line joining the outer canthus of the eye to the top of the ear, and including the upper eyelid, may predict central nervous system (CNS) and/or ocular involvement (4) (Figure 1A). This association may be the consequence of the common origin from the prosencephalon of the frontonasal prominence, meninx, and encephalon. The forehead, nose, philtrum, and primary palate derives from the frontonasal prominence (5). Indeed, an early mutation in GNAQ gene in the prosencephalon may cause a complex vascular anomaly distributed in the skin, eye, and the CNS. Recently, two cases of SWS due to GNA11 have been described (6, 7). A somatic GNAQ mutation is present in SWS (8–10).

*PIK3CA related overgrowth spectrum*. CMs may be associated with segmental overgrowth and sometimes with skeletal anomalies. Somatic mutations in the phosphatidylinositol-4,5-bisphosphate 3-kinase, catalytic subunit alpha (*PIK3CA*) gene have been identified in patients affected with various syndromes characterized by vascular anomalies and segmental overgrowth. Initially, the term PROS included Fibroadipose hyperplasia or overgrowth, hemihyperplasia multiple lipomatosis, congenital lipomatous overgrowth, vascular malformations, epidermal nevi, scoliosis/skeletal and spinal syndrome, macrodactyly, fibroadipose infiltrating lipomatosis (CLOVES), and megalencephaly-capillary malformation (MCAP) (11). Subsequently, other entities have been added to this spectrum. Indeed, PIK3CA mutations have been described in 21 patients with Klippel–Trenaunay syndrome (KTS) (12), in capillary malformation of the lower lip, lymphatic malformations of the face and neck, asymmetry, and partial or generalized overgrowth (CLAPO) syndrome (13), and in diffuse capillary malformation with overgrowth (DCMO) (14).

CM in PROS has peculiar characteristics: the color is generally violaceous with geographic borders (Figure 1B). The localization depends on mosaic distribution but some areas are more frequently involved: the lateral aspect of the thigh in KTS (Figure 1C), the philtrum in MCAP (Figure 1D), the hip in CLOVES (Figure 1B), and the lower lip in CLAPO (Figure 1E). In MCAP and DCMO, CM is diffused with reticulated pattern and checkboard distribution (Figure 1F). CM due to PIK3CA mutations is commonly associated with lymphatic and/or venous malformations, which may manifest as grouped hyperkeratotic lymphangiectasias and prominent superficial veins (Figures 1B,C).

**Figure 2:**
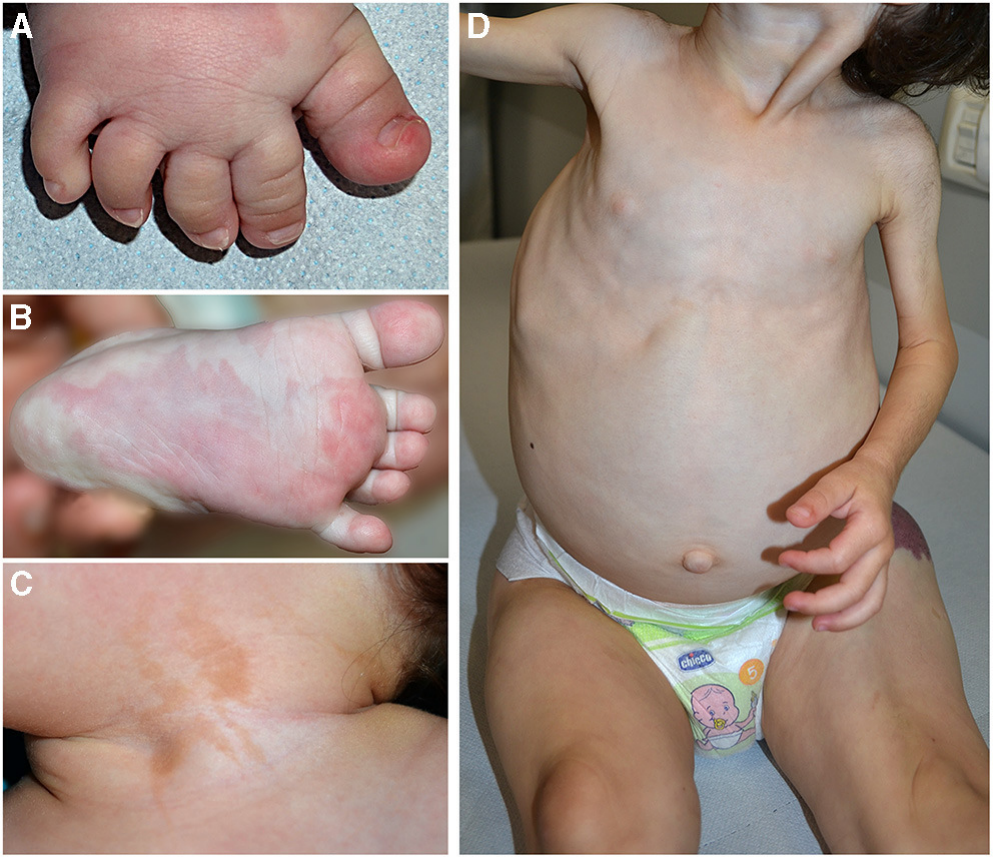
Pseudosyndactyly **(A)**, sandal gap, and capillary malformation in a patient with PIK3CA-related overgrowth syndrome **(A)**. Triangular shape of the left foot **(B)** and epidermal nevus of the neck in the same patient **(C)**. Plus/minus sign, overgrowth of lower limbs in contrast with upper limbs hypoplasia in a child affected with CLOVES **(D)**.

Clinical features frequently associated in PROS are pseudosyndactyly (Figure 2A), sandal gap, and triangular shape of the feet (Figures 2A,B). Epidermal nevus is a clinical sign of CLOVES syndrome, but it may be observed also in other PROS (11) (Figure 2C). In patients with MCAP, CM is associated with macrocrania. CLOVES and MCAP frequently manifest progressive overgrowth of lower limbs in contrast with upper limb hypoplasia (plus/minus sign) (Figure 2D).

Generalized overgrowth conditions associated with CM have increased risk to develop Wilms' tumor. However, the occurrence of malignancy in these patients is less frequent than in Beckwith–Wiedemann syndrome. Peterman et al. reported that Wilms tumor screening is recommended only in patients with hemihypertrophy and may be required in MCAP (15).

**Figure 3:**
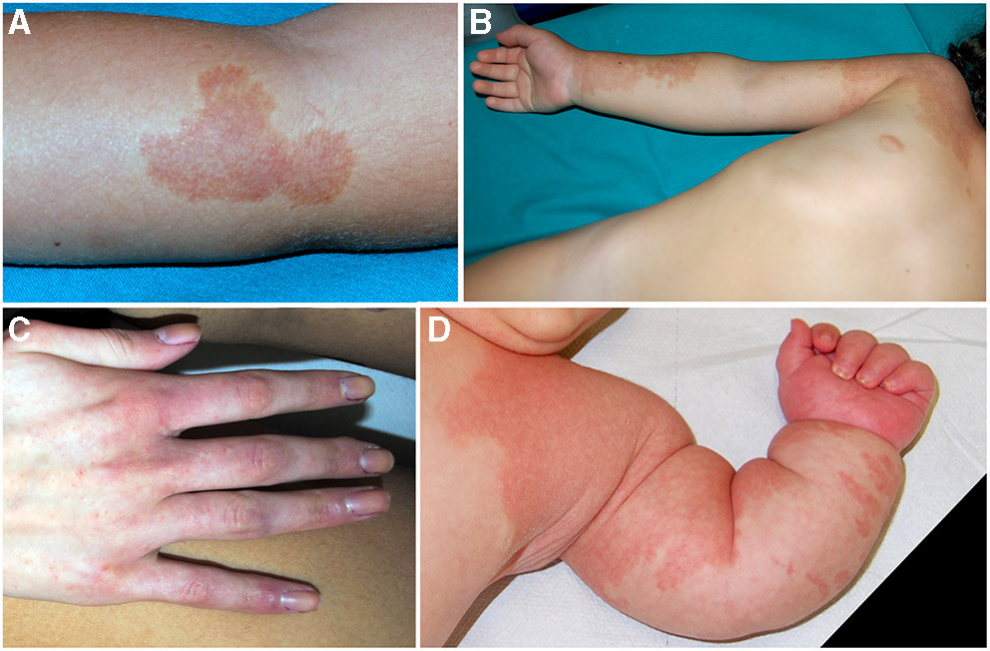
Polycyclic vascular patch characterized by an anemic halo in a patient with CM-AVM **(A)**. Pink-red slightly pigmented capillary malformation (CM) of the right upper limb **(B)**. Pinpoint confluent telangiectasias in a patient with EPHB4 mutation **(C)**. Eight-month-old infant affected with Parkes–Weber syndrome with multiple CM and progressive overgrowth of the upper limb **(D)**.

*Capillary malformation arteriovenous malformation (CM-AVM)*. CM-AVM is a distinct entity; it is part of the arteriovenous malformations that derive from an altered vascular morphogenesis causing malformed arteries, veins, and capillaries with arteriovenous communications. CM-AVM has a prevalence of 1/100,000 (16) characterized by several round-to-oval CM with a diameter of few centimeters and a peculiar anemic halo (Figure 3A). The color of the lesions is pink, but they become hyperpigmented with time mimicking caffè-au-lait spots (Figure 3B). Indeed, on dermoscopy, CM lesions appear telangiectasic with a hyperpigmented background detectable after pressure (17). One-third of the affected patients presents arteriovenous fistulas (AVFs) in muscles, soft tissue, or CNS (18). These cases may carry pinpoint lesions that resemble common teleangiectasia, characterized by a typical whitish halo (Figure 3C), which is not present in Hereditary Hemorrhagic Teleangiectasia (HHT).

CM-AVM is an autosomal dominant syndrome due to mutations in *RASA1* or *EPHB4*. Pathogenic variants in *RASA1* and *EPHB4* have been identified in ~50 and 25% of the cases, respectively (18, 19). Germline loss-of-function mutations in EPHB4 causes a second form of CM-AVM (CM-AVM2) deregulating RAS-MAPK signaling (19).

Amyere and colleagues first described mutations in *EPHB4* and proposed to distinguish between CM-AVM1 and CM-AVM2 caused by mutations in *RASA1* and *EPHB4*. Telangiectasias distributed on hands, lips, and upper thorax are associated with mutation in EPHB4.

In patients in whom mutations have not been identified, the disease may be explained by genomic gene rearrangements, intronic mutations or, in regulatory regions, epigenetic factors or mosaicism (20). In the last condition, severity of the phenotype is not related to the level of mosaicism identified in blood (20). Moreover, the extreme intra-familiar variability of the syndrome is due to a two-hit mechanism as confirmed by the identification of germline and lesional somatic mutations in *RASA1* (21).

Mutations in *RASA1* may cause Parkes–Weber syndrome (PWS), which is part of the spectrum of RASA1-related diseases. In addition to multiple small CMs, patients affected with PWS present large CM associated with AVFs and progressive overgrowth of the involved limb (Figure 3D).

The fast-flow vascular malformations may develop in the CNS, muscles, bones, and skin. Venous dilation in PWS patients is secondary to blood hyper-flow and not to venous valve insufficiency. For asymptomatic patients or for those with minor symptoms, conservative approach is recommended. Treatment decision should be evaluated by a multidisciplinary team. Complete excision is not usually possible, but in selected patients, catheter embolization may be combined with surgery (22). Multiple AVFs causing high-output cardiac failure require embolization and surgery.

### Venous Malformations

**Figure 4:**
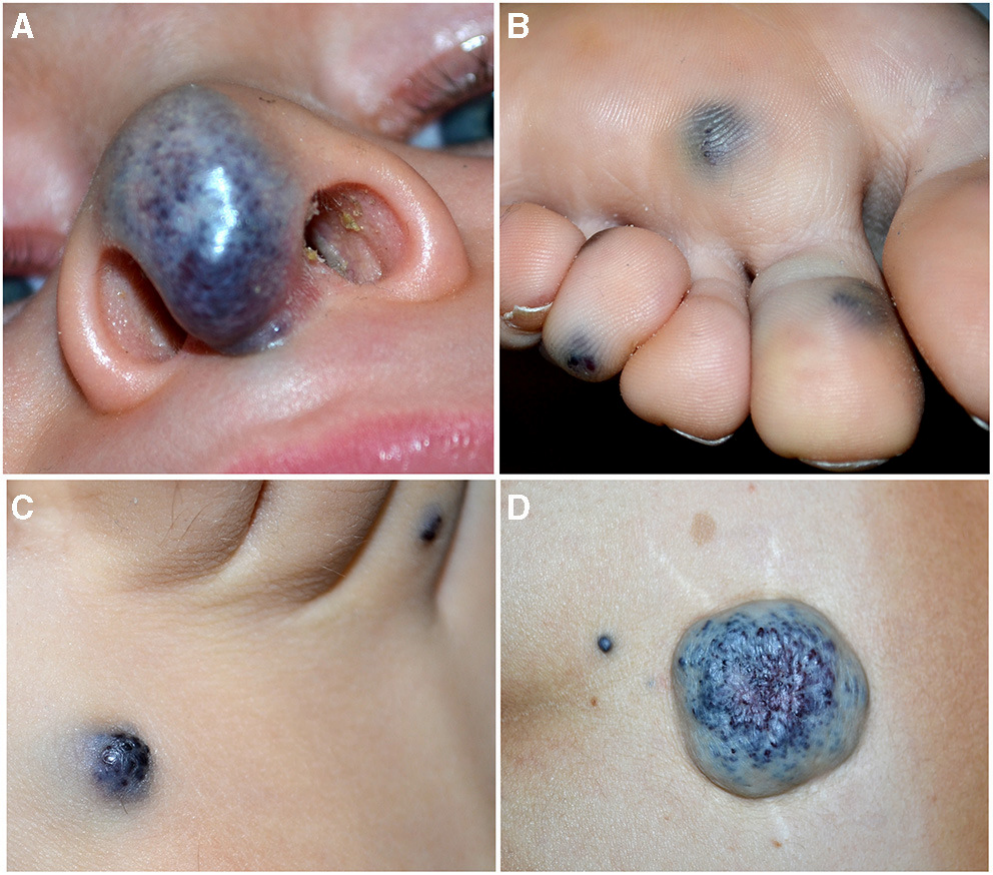
Soft blue malformation of the tip of the nose in a toddler with a solitary venous malformation (VM) **(A)**. Multiple small blue cutaneous VMs of the plantar **(B)** surface in a patient affected with blue rubber bleb nevus syndrome. Blue rubber bleb of the dorsal surface of the foot **(C)** and “dominant” congenital cutaneous VM of the lumbar area in the same patient **(D)**.

*Venous malformation (VM)*. It is the second common vascular malformation with an incidence of 1–5 in 10,000 births. They are slow-flow lesions, generally cutaneous unifocal and sporadic. They may be superficial or profound; when visible on the skin, they appear blue, soft, and compressible (Figure 4A). About 1–2% of VMs are multifocal and familial, and occur with a two-hit mechanism. They can develop in any part of the body, and the gastrointestinal tract is the most frequent visceral localization, which may manifest with hemorrhage and anemia.

*Blue Rubber Bleb Nevus Syndrome (BRBNS). First* described by Gascoyen in 1,860, it is a rare sporadic condition caused by somatic double (cis) mutations in *TEK* gene (23). Patients present several VMs in the skin and/or visceral organs. BRBNS is characterized by gastrointestinal grape-like lesions visible on endoscopy, a “dominant” cutaneous or subcutaneous VM (>10 times the other visible lesions), and numerous other small (1–2 cm) cutaneous VMs. Skin lesions are blue to purple in color, compressible, often hyperkeratotic, and mainly located in the palmoplantar areas, and they increase in size and number with time (24) (Figures 4B–D). Anemia due to recurrent gastrointestinal bleeding is the major complication of this syndrome.

### Lymphatic Malformations

**Figure 5:**
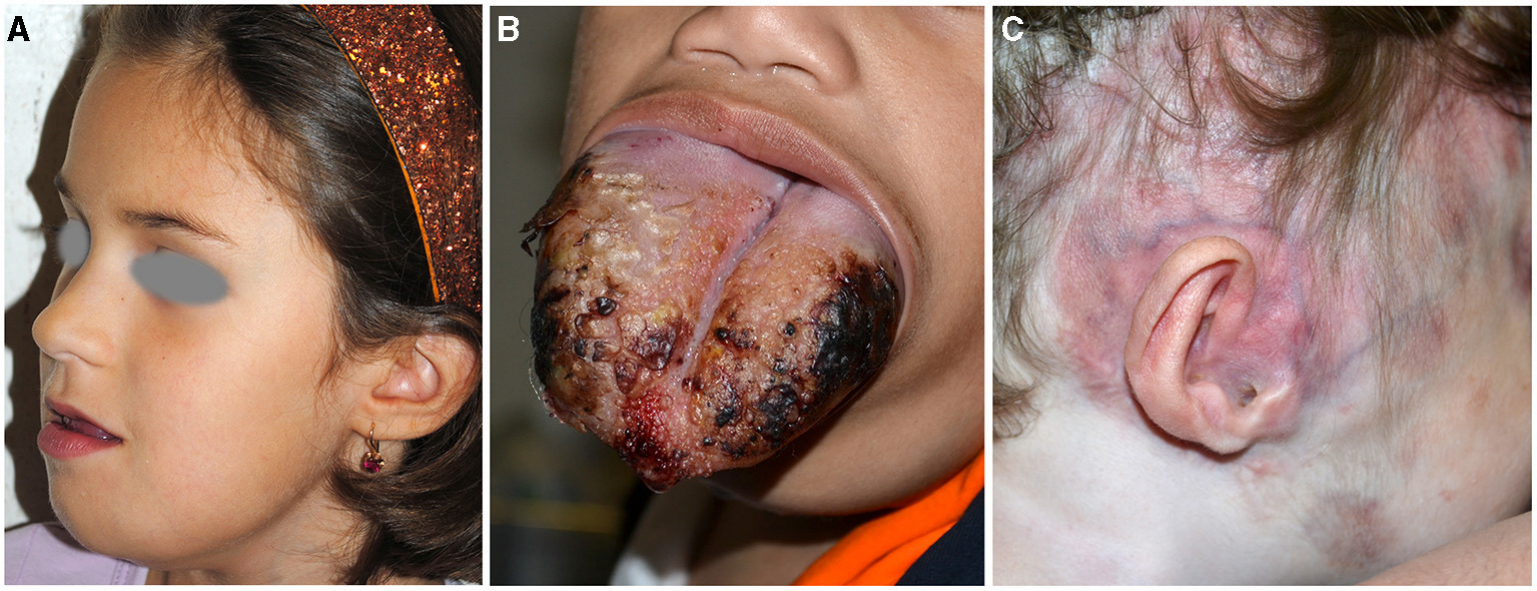
Microcystic lymphangioma involving the tongue, the submandibular area, and the chin causing macroglossia, deformity, and sialorrhoea **(A)**. Microcystic lymphangioma of the tongue in an 18-month-old patient treated with tracheostomy and percutaneous endoscopic gastrostomy for dysphagia and respiratory distress **(B)**. CM and deformity of the auriculotemporal area due to osteolysis in a child affected with Gorham–Stout disease **(C)**.

*Micro and macrocystic lymphatic malformations (LM)*. They are congenital slow-flow malformations of lymphatic vessels with an incidence of 1.2–2.8 per 1,000 births. They are distinguished in micro-, macrocystic, or mixed depending on the dimension of the cysts, and occur within the first 2 years of life in 90% of the cases (25). They manifest as subcutaneous masses suddenly appearing or worsening after a minor infection, or with grouped transparent or blood-filled vesicles on the skin or oral mucosa. Disfigurement or compression of nearby structures with functional damage are the most important complications. In particular, 75% of LM is localized in the head and neck, with a predilection for submandibular region and parotid (Figure 5A) (26). Microcystic lymphangioma may involve the tongue and oral cavity causing macroglossia and related complications (sialorrhoea, dysphagia, and respiratory distress) (Figure 5B). The majority of the patients are initially asymptomatic; however, in more severe cases, tracheostomy and percutaneous endoscopic gastrostomy are needed to guarantee airway patency and adequate nutrition.

Excision of these malformations is usually not feasible due to the absence of clear margins, high risk of potential disfigurement, and functional impairment. Sclerotherapy is mostly indicated for macrocystic lesions, as isolated procedure or preceding surgery (27). In fact, surgical treatment has a high recurrence rate and combined therapy is required in most of the cases.

Gorham–Stout disease (GSD) and generalized lymphatic anomaly (GLA) are very rare LMs potentially involving all body sites, including the skeleton.

GSD is characterized by the presence of bone infiltration by microcystic lymphatic malformation with osteolysis and cortical bone resorption documented through computed tomography (CT). Patients manifest pathological fracture and pain and may have scoliosis and lymphorrhea and deformities (Figure 5C).

GLA is a multi-organ diffuse disease. Respiratory distress and bone fractures are the major complications in these patients. Pleural and pericardial effusion, rib and vertebral involvement, and young patient age are associated with a worse prognosis in GLA and GSD. Other localization may be the spleen, the peritoneum, and the gastrointestinal tract.

### Vascular Tumors

IH is a benign vascular tumor characterized by three phases. It generally occurs after birth with a high proliferation during the first months of life, followed by a stabilization of the lesion at ~9 months of age and involution within 4–6 years of life (3).

**Figure 6:**
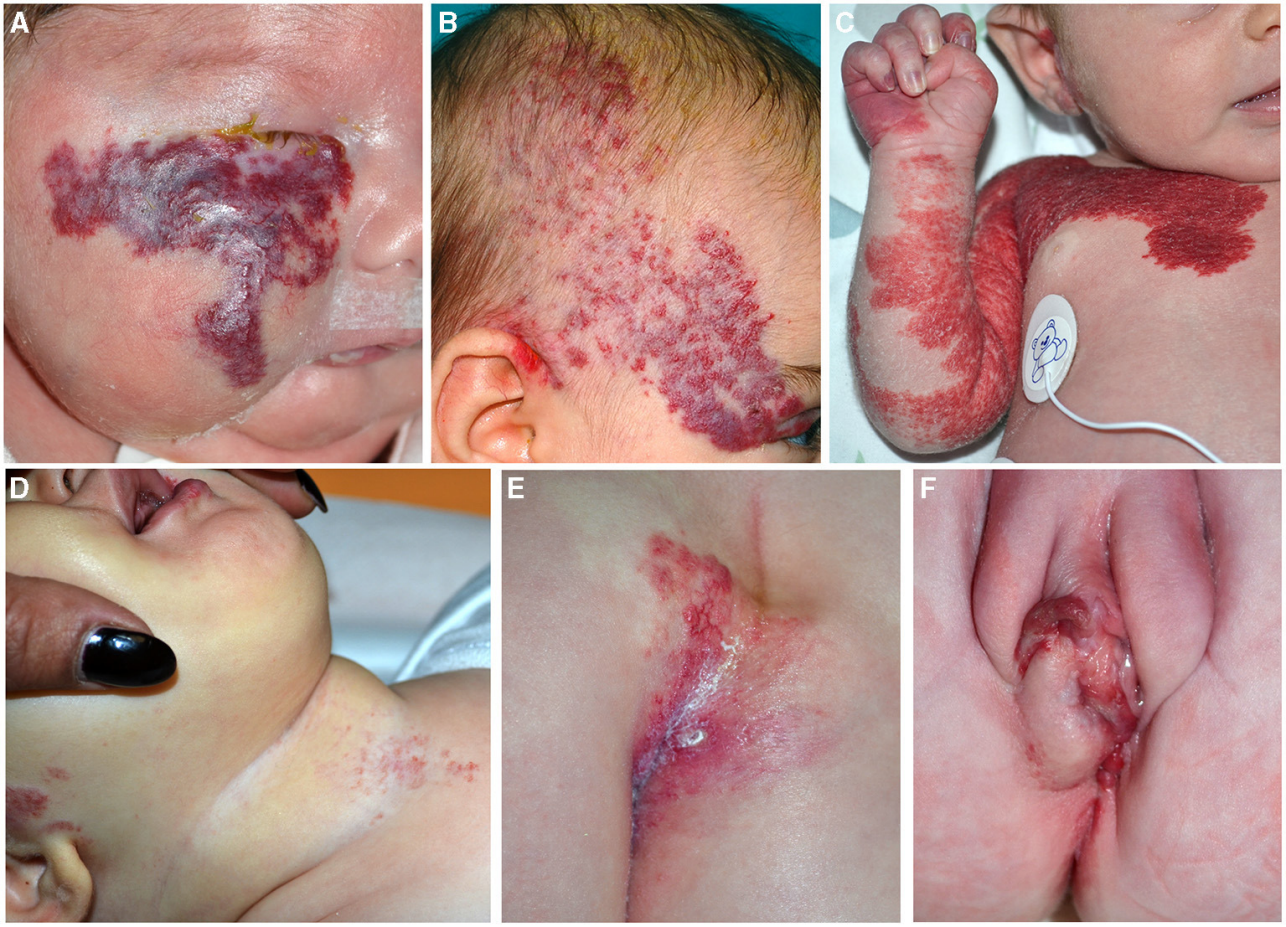
Segmental infantile hemangiomas (IH) of the face **(A,B)**; the lesions have a diameter of than 5 cm and must be investigated for PHACE syndrome **(A)**. Segmental IH of the upper part of the trunk and the upper limb in a neonate **(C)**. Segmental IH is on the beard area in an infant with obstruction of the upper airways **(D)**. Segmental lumbar and sacral IH associated with myelopathy **(E)**. IH of the vulva area associated with genital anomalies and imperforate anus **(F)**.

They may be cutaneous, superficial, deep or mixed, and/or extracutaneous, focal, or multifocal. IH diameter ranges from a few millimeters to more than 5 cm (large lesions). Focal lesions have a round shape, while segmental IHs may involve one or more segments in a broad anatomic territory of skin (Figure 6A). Large segmental IHs may be associated with malformations of underlying structures in the same developmental field (3). Indeed, depending on the localization of the tumor, different syndromes have been described such as PHACE and LUMBAR/SACRAL/PELVIS.

PHACE syndrome includes posterior fossa malformations, hemangioma, arterial anomalies, coarctation of the aorta/cardiac defects, eye abnormalities, and sternal malformations. Not all these features are necessarily present to establish the diagnosis, which is based on specific criteria established in 2009 and modified in 2016 (28, 29). The IH should be large (more than 5 cm) on the face (Figure 6B), but the syndrome may be present even with an IH of the upper part of the trunk and the upper limb or in the absence of IH if major criteria are present (Figure 6C). The syndrome has a prevalence of 2–3% of all IHs and 20–30% of large segmental IHs of the face (30). The female/male ratio is 9:1. Brain magnetic resonance imaging (MRI) findings lead to the stratification of low, intermediate, and high risk of a cerebrovascular accident in patients with PHACE syndrome (29). Based on the localization of the IH, there is a different risk of extracutaneous involvement: the eye and brain in frontotemporal and frontonasal IH, and the heart in mandibular lesions (31).

Other associated clinical features are headache, endocrine abnormalities, and auditory and dental anomalies. The beard pattern has been identified by the presence of IH in the right and left preauricular areas, lower lip, chin, and anterior neck. When segmental IH is distributed on the beard area, larynx can be involved, causing potentially life-threatening obstruction of the upper airways (Figure 6D). More rarely a subglottic IH may occur as a persistent croup-like illness in the absence of a cutaneous IH (32). In 50% of the patients affected with subglottic his, four or more areas were involved by the tumor, whereas 4% of cases had IH in one or two areas of the beard. The most frequently involved skin area in patients with subglottic IH is the mandibular (median) area (63% of the cases in asymptomatic patients) (33, 34). Cutaneous IHs associated with subglottic involvement were significantly more telangiectatic than raised superficial lesions (33). Moreover, IHs of the beard area may be considered distributed in a segmental pattern and they are potentially associated with PHACE syndrome, as reported by Metry et al. (35). In addition, PHACE syndrome may be associated with subglottic IH even in the absence of beard IH (36).

Lower body IH and other skin defects, urogenital anomalies and ulceration, myelopathy, bony deformities, anorectal malformations, and arterial and renal anomalies (LUMBAR) syndrome occur when a large segmental IH is localized in the lower part of the body in association with extracutaneous regional malformations (Figures 6E,F). This acronym is now considered more comprehensive of the malformations that can be associated with IH in the lower part of the body, replacing the terms spinal dysraphism, anogenital, cutaneous, renal and urologic anomalies, associated with an IH of lumbosacral localization (SACRAL) and perineal hemangioma, malformations of external genitalia, lipomyelomeningocele, vesicorenal abnormalities, imperforate anus and skin tag (PELVIS) syndrome (37).

The most common localization of IH is the sacral region followed by the lumbar, perineum, and the lower limbs. Segmental IH located in the sacral region may be associated with urogenital and anorectal malformations, while IH in the lumbar area may be associated with myelopathy. Ulceration is frequent when more than three regions are affected (37).

**Figure 7:**
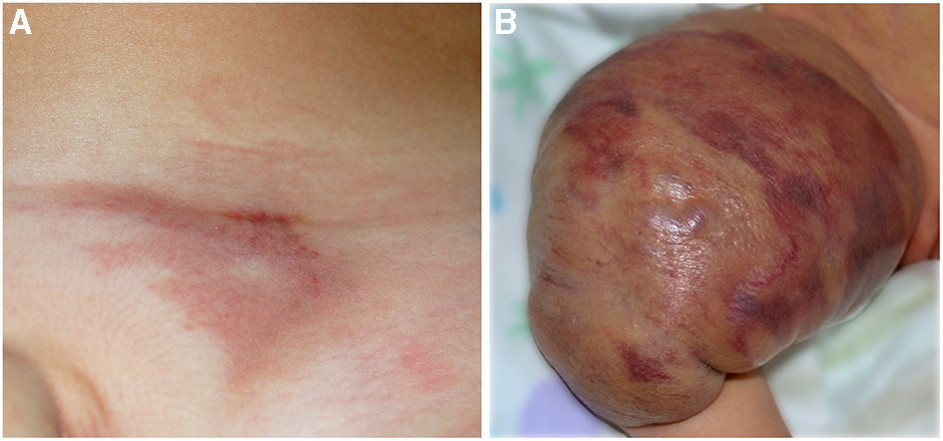
Red vascular plaque of the pubic area in a toddler **(A)**; the lesion is painful and warm. Voluminous vascular tumor characterized by red brilliant purpuric color associated with the Kasabach–Merritt phenomenon in a newborn **(B)**.

*Tufted angioma (TA)*. It is a benign vascular tumor whose name derives from the “tufts of hypertrophied endothelial cells” (38) in the dermis with a cannonball distribution. TA may be congenital or acquired in the first years of life. It is usually a solitary red purple plaque or nodule, but multifocal cases have been reported (Figure 7A). Most of the cases are asymptomatic; pain may be present. In a minority of the cases, Kasabach–Merritt phenomenon (KMP) may be associated. It is frequently distributed in the head and neck or in the upper trunk, but other localizations are described. Even if spontaneous regression may occur, persistent lesions are frequently painful and treatment is needed (39).

*Kaposiform hemangioendothelioma (KHE)*. KHE is a rare locally aggressive vascular tumor characterized by the presence of spindle cells with a positive staining for D2-40 lymphatic marker. On histology, KHE has an infiltrative behavior and may affect tissues, muscles, and bones with possible visceral involvement (mediastinum and retroperitoneum). The onset is at birth or in infancy presenting as a reddish plaque or multiple nodules (Figure 7B). In the majority of cases (42–71%), KHE is associated with the KMP (40). It is a life-threatening consumptive coagulopathy causing severe thrombocytopenia, together with hypofibrinogenemia (41). When KMP occurs, the lesion becomes swollen, hot, and purpuric with a relevant enlargement. Combined treatments are warranted for KMP, and platelet transfusion should be performed only in case of bleeding or prior to surgery (40). Apart from KMP, other complications are possible in KHE: musculoskeletal disorders, lymphedema, and compression of vital structures (40). The diagnosis is made on histology, but MRI is necessary to define margins and deepness of the tumor (42).

## Discussion

Consultations for vascular birthmarks are very common in dermatology, given the high rate of vascular lesions in the neonatal period. The vast majority of the cutaneous manifestations are transient or benign (salmon patch, nevus simplex, and stork bite) and reassuring the parents is sufficient. The assessment of the patients should start from a complete clinical history (family, pregnancy, and personal history of the mother and the child). Indeed, some vascular malformations such as multifocal VM and CM-AVM may recur in the family, while IH is associated to low birthweight, *in vitro* fertilization, prematurity, and twin pregnancy (3, 43, 44).

It is very important to determine if the vascular anomaly was present at birth or developed in the first days/weeks of life. Vascular malformations are present at birth, but sometimes deep venous and lymphatic malformations may become visible after birth because of thrombosis or swelling, respectively. On the other hand, IHs occur in the first weeks of life sometimes preceded by an anemic patch, while congenital hemangiomas are always present at birth and do not increase in size. In addition, congenital hemangiomas do not have risk factors like IHs. If precise documentation of the lesion at birth is not available, it is useful asking the parents to show photographic images of the newborn.

**Figure 8:**
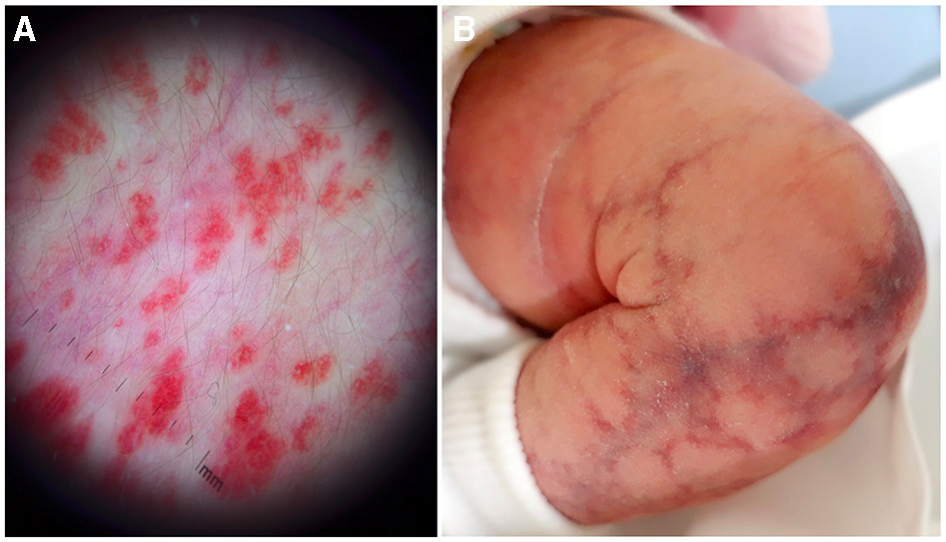
Dermoscopy of a flat infantile hemangioma shows a micro-papular pattern and allows to differentiate it from capillary malformation **(A)**. Cutis marmorata telangiectatica congenital with the typical reticulated linear atrophy **(B)**.

In the presence of a superficial vascular anomaly, the differential diagnosis between vascular malformations and vascular tumor is easy for an expert dermatologist, according to the ISSVA classification updated in 2018. Rarely, IHs may present as a flat vascular patch; dermoscopy highlights the micro-papular pattern of the lesion and allows to differentiate it from CM (Figures 6B, 8A). CMs appear as a flat homogeneous red patch with sharp margins. VMs manifest as soft blue masses on the skin or mucous membranes that typically enlarge when in declive position. Microcystic lymphatic malformation may be superficial, emerging on the skin as grouped vesicles with transparent or hematic content. These lesions may ooze or bleed especially in summer.

The extension of the vascular malformation may correlate with severity of the disease. Indeed, the wider is the diffusion of the malformation, the earlier must be the mutation responsible for the phenotype, with a wider involvement of tissue and organs. In general, localization of vascular birthmark on the head may be associated with cerebral involvement. The main variables that determine the phenotype in mosaic disorders is the mutation, the normal function of the gene, the timing of the mutation during embryogenesis, and the destiny of the mutated cell. Indeed, different clinical results may be produced by an identical genetic defect (45).

Clinical examination should always be complete and not limited to the vascular anomaly. Indeed, in particular with CM, many other cutaneous or extracutaneous signs should be investigated. The presence of other nevoid lesions may orientate the diagnosis: an epidermal nevus associated with CMs may suggest a PROS, while a large nevus spilus may suggest a phacomatosis spilorosea.

Macrocephaly together with CMs may be a clue for the diagnosis of megalencephaly-CM or PTEN hamartoma tumor syndrome. Pseudosyndactyly, sandal gap sign, and limb overgrowth may be associated with CM and overgrowth in PROS. The presence of a vascular anomaly, segmental IH or a CM, in the lumbar and sacral region suggests the presence of associated malformation: bone deformities, anorectal malformations, lipomatous masses, spinal dysraphism, urogenital, and renal malformations.

Doppler-ultrasound is the examination of first choice for suspected deep vascular anomaly (46). Indeed, this non-invasive technique usually allows the radiologist to identify a vascular lesion, to distinguish between tumor and malformation, and to confirm a slow-flow or fast-flow vascular anomaly. Nevertheless, newborns and infants do not collaborate and cry, producing a lot of artifacts. For this reason, based on our clinical experience, we tell the mother that the baby should be fasted and to breastfeed or to give the feeding bottle in the waiting room to help the newborn to sleep or during the exam in order to improve the quality of the examination.

Differential diagnosis of deep vascular lesions is between vascular tumors and malformations but also with non-vascular malignant tumor. For this reason, when the clinical history, together with the clinical characteristics, and the Doppler-ultrasound are not diagnostic, a biopsy is mandatory. In some cases, the surgeon requires magnetic resonance imaging before the procedure in order to investigate the margins of the lesion and its relationship with surrounding structures (47).

ISSVA classification represents an important step forward that has provided a common language for the scientific community dealing with vascular anomalies. Nevertheless, with the aim of schematizing and relate different clinical pictures to a single recognized entity, there is a risk of oversimplifying. It is the case of CMs that have been defined as slow-flow CMs but are included in the definition of CM-AVM, a disease that is known to be a fast-flow vascular malformation. CMs in CM-AVM are usually multiple, and they have a peculiar clinical feature: small red pink color patches with round margins and a white anemic halo, and they are warm. Sometimes, it is possible to appreciate a bruit or a trill underneath. The difference between slow-flow CMs and CMs in CM-AVM has been demonstrated by Valdivielso-Ramos M et al., who suggest that they histologically and immunohistochemically resemble an incipient arteriovenous malformation (48). In addition, CMs in CM-AVM are familial, while simple CMs are not.

Moreover, CM due to GNAQ mutation has a different appearance from CM observed in PROS. Indeed, the latter exhibits a more geographical margin, a dark red color, and may present lymphangiectasias on the surface and an underlying prominent venous reticulum. Normally, hypertrophy of soft tissue due to GNAQ mutation, if present, is lower than the one due to PIK3CA mutation, which is typically progressive. When CM is located in the frontonasal area, the eye and the pia mater may be involved due to an early mutation in the prosencephalon. The patient may be affected with SWS and presents epilepsy and/or glaucoma. Controversies are reported in the literature on when to carry out brain MRI in asymptomatic patients who present CM potentially associated with SWS. An exhaustive examination is possible only after the age of 1 year due to maturation of brain. In the absence of neurological symptoms, it is recommended to postpone brain MRI in general anesthesia with gadolinium to the second year of life (49). Brain MRI during sleep is possible in the first months of life but normal results do not rule out SWS.

CMs in phacomatosis spilorosea have a characteristic pink color and have been recently associated with mutation in *PTPN11* (6). Cutis marmorata telangiectatica congenita is actually classified as a CM, but has a reticulated pattern with a peculiar linear atrophy and no mutations have been yet identified in this malformation (Figure 8B). In addition, the evolution is completely different: ulceration is possible, but the lesions improve enormously in the first 6 months of life, while CMs are usually stable

VMs are distinguished into common VM, familial CutaneoMucosal VM (VMCM), glomuvenous malformation (GVM), Blue Rubber Bleb Nevus syndrome (BRBNS), and cerebral cavernous malformation (CCM). In case of a venous malformation, accurate family history to rule out a familial condition is mandatory as well as a full blood count and fecal occult blood test. Indeed, single cutaneous lesions have also been recently reported in patients with intestinal involvement, which may manifest with bleeding, requiring an emergency treatment (50).

Moreover, VM is generally present at birth, but multiple lesions may develop later in life due to a two-hit mechanism in the *TEK* gene (23).

GMVs are easily diagnosed; they manifest with multiple cutaneous blue macules or papules, sometimes grouped in patches and painful on pressure. They are due to a mutation in the *glomulin* (*GLMN*) gene.

BRBNS may be suspected in the presence of a “dominant” congenital cutaneous lesion associated with multiple compressible, often hyperkeratotic, small skin lesions frequently located on palmoplantar surfaces. Additional VMs appear during life and gastrointestinal lesions may cause severe anemia.

Lymphatic malformations are localized in the head and neck in 75% of cases and frequently involve deep and vital structures. They should be suspected in patients who manifest sudden swelling of the neck or parotid in the first years of life. A careful observation of the oral mucosa may identify typical grouped vesicle of the microcystic lymphangioma. In more severe cases, macroglossia and obstructive respiratory symptoms are present. Diagnosis may be easily confirmed with ultrasound but magnetic resonance is mandatory to study the extension of the malformation and its relation with the neighbor structures. In case of obstruction of the upper airways, laryngoscopy is mandatory in order to evaluate the percentage of obstruction and to discuss the necessity of a tracheostomy.

Concerning vascular tumors, it is known that 10–12% of IHs may require treatment due to ulceration, life threatening complications, functional damage, and permanent disfigurement.

In case of a segmental and large IH, it is mandatory to rule out associated malformations when the tumor is located in the head, neck, arm and upper trunk or in the lower part of the body. When PHACE syndrome is suspected, brain MRI, ophthalmologic evaluation, and echocardiography should be performed. In case of lower body large and segmental IH, or IH on the median line, LUMBAR syndrome should be ruled out with spinal and pelvic MRI and abdominal ultrasound (51). In addition, in newborns with large IH, or with multiple liver IHs, it is necessary to investigate hypothyroidism.

Congenital hemangiomas are usually easily distinguished due to their clinical characteristics. They are frequently located on head and neck and extremities. RICH has a dome shape with a pale rim and a central depression while NICH is slightly elevated with a blue hue covered by thick telangiectasias and with a peripheral pallor. They are large at birth and never increase in size; NICH stably persists, while RICH is rapidly involved within the first 18 months of life or partially regresses (PICH). Congenital hemangiomas are not a sign of a complex disorder; however, they should be considered in differential diagnosis with other vascular tumors and neoplastic disorders.

When an angiomatous mass of a newborn is associated with thrombocytopenia, KMP should be suspected. KMP occurs in TA and KHE that may be congenital or appear after birth. Rarely, RICH may be associated with a transient thrombocytopenia. Patients with TA or KHE should be regularly investigated in the first 6 months of life. In KMP, platelets are very low, fibrinogen is low, and D-dimers are very high, but it has been a common experience of the experts that these patients do not manifest spontaneous hemorrhages as expected based on the laboratory values. Moreover, in KMP, platelets should never be transfused with the exception of bleeding or immediately prior to surgery. Steroids and acetylsalicylic acid should be immediately started, and biopsy is suggested in order to confirm the diagnosis and to start II level treatment with sirolimus or vincristine. KMP is present only in the first months, and then, after treatment and regression, the vascular tumor may present as a pseudo-CM, with telangiectatic streaks and swelling or with a scleroderma-like plaque.

TA usually presents as a reddish-brownish single or multiple patch sometimes infiltrated with hyperidrosis or hypertrichosis. KHE is frequently deeper and may infiltrate the mediastinum and retroperitoneum sometimes involving the skin as a reddish-violaceous plaque or a voluminous mass.

In the recent years, many advancements have been achieved in the understanding of the molecular mechanism and genetic causes of vascular anomalies. While mutated genes in familial vascular anomalies are vascular cell-type specific, genes mutated in sporadic vascular malformations are widely expressed in all tissues. The RAS/MAP/ERK and the PI3K/AKT/mTOR signaling pathways are regulators of cell proliferation, migrations, and apoptosis, and are involved in oncogenesis. Mutation in these two pathways is responsible for the vast majority of vascular malformations (52).

It is now clear that multiple entities are part of wide spectrum of possibilities depending on the mutated gene, site, and timing of the mutation. For example, PROS includes different phenotypes with variable severity, from macrodactyly to CLOVES syndrome. Moreover, identification of causative genes of the vast majority of vascular malformations has opened up the possibility of repurposing anticancer molecules as new target therapies for vascular malformations. Sirolimus, a mTOR inhibitor, has shown promising results in the management of complicated vascular anomalies (53, 54). In addition, new molecules such as Alpelisib, a PIK3CA inhibitor, and Miransertib, an AKT inhibitor, are nowadays the subject of clinical trials (55). Moreover, MEK inhibitors and Vemurafenib have restored vascular anomalies in a zebrafish model of EPHB4, BRAF, and MAP2K1 mutations, respectively (56).

Biopsy of superficial lesions is a very simple and fast procedure, but it requires general anesthesia in children below the age of 6 years. On the contrary, in the first 2 years of life, the child is not fully aware of what happens around him and it can be easy to collect a few millimeters of a sample of affected tissue. For this reason, we suggest, in more severe cases who may be candidates for a target therapy, for the child to immediately undergo genetic testing. Recently, cell-free DNA next-generation sequencing liquid biopsy, a technique derived from oncologic settings, has demonstrated to be an effective tool for KTS diagnosis, opening a new possibility for other mosaic vascular anomalies as a non-invasive diagnostic approach (57). In addition, in patients with phenotype involving the head or the brain, NGS from buccal swab demonstrated a sensitivity comparable to NGS from tissue biopsies. In these patients, the detection rate of the pathogenic or likely pathogenic mutation from buccal swab increases to 57% (58). Altogether, these new techniques will allow to perform more diffusely genetic testing in newborns and infants, in order to early detect the pathogenic mutations responsible for the phenotype.

In conclusion, cutaneous birthmarks may be an important clue for the diagnosis of complex and syndromic vascular anomalies but is often underestimated. They represent a phenotypic and genotypic heterogeneous wide group of frequently disabling disorders with potentially severe and life-threatening complications. Even if recent advancement in genetics allowed better understanding of the pathogenesis of these diseases and their classification, controversies are still present and management is not yet fully standardized. Finally, in selected cases, genetic testing should be rapidly performed in order to confirm the diagnosis and to offer genetic counseling; identification of the mutated gene may offer the opportunity to undergo target therapy and to be enrolled in clinical trials. For these reasons, patients should be promptly referred to a multidisciplinary reference center for appropriate management and follow-up.

## Author Contributions

AD and ME: conceptualization, project administration, and supervision. AD, GP, MZ, RA, CCa, AC, CCe, MD, SB, and MR: investigation. GP, MZ, RA, and MR: visualization. AD, CCa, and ME: writing—original draft. AD, GP, MZ, RA, CCa, AC, CCe, MD, SB, MR, and ME: writing—review and editing. All authors contributed to the article and approved the submitted version.

## Conflict of Interest

The authors declare that the research was conducted in the absence of any commercial or financial relationships that could be construed as a potential conflict of interest.

## Publisher's Note

All claims expressed in this article are solely those of the authors and do not necessarily represent those of their affiliated organizations, or those of the publisher, the editors and the reviewers. Any product that may be evaluated in this article, or claim that may be made by its manufacturer, is not guaranteed or endorsed by the publisher.
